# A Randomized Controlled Trial: Regenerative Effects, Efficacy and Safety of Erythropoietin in Burn and Scalding Injuries

**DOI:** 10.3389/fphar.2018.00951

**Published:** 2018-10-31

**Authors:** Christina I. Günter, Hans-Günther Machens, Felicitas P. Ilg, Alexander Hapfelmeier, Wolfgang Jelkmann, Silvia Egert-Schwender, Shibashish Giri, “EPO in Burns” Study Group, Augustinus Bader

**Affiliations:** ^1^Clinic for Plastic Surgery and Hand Surgery, Klinikum rechts der Isar, Technical University of Munich, Munich, Germany; ^2^Institute of Medical Informatics, Statistics and Epidemiology, Klinikum rechts der Isar, Technical University of Munich, Munich, Germany; ^3^Institute of Physiology, University of Lübeck, Lübeck, Germany; ^4^Münchner Studienzentrum, Klinikum rechts der Isar, Technical University of Munich, Munich, Germany; ^5^Applied Stem Cell Biology and Cell Technology, Biomedical and Biotechnological Center, Leipzig University, Leipzig, Germany; ^6^Department of Plastic Surgery and Hand Surgery, University Hospital Rechts der Isar, Technische Universität München, Munich, Germany; ^7^Institute for Cell Techniques and Applied Stem Cell Biology, Leipzig University, Leipzig, Germany

**Keywords:** erythropoietin (EPO), burn injuries, regenerative medicine, wound healing, randomized clinical trial

## Abstract

In adult’s burn injuries belong to the top 15 causes of injury. Annually more than a million patients receive specialized treatment. Improving burned patients’ outcomes is still a challenge. Effects of erythropoietin (EPO) are reported to be pro-angiogenic, pro-regenerative, anti-inflammatory, immunomodulatory and hypoxia/ischemia protective. Study objectives were to demonstrate cytoprotective and regenerative effects of EPO in burned patients in terms of improved wound healing, reduced morbidity and mortality. This was a prospective, placebo-controlled, randomized, double-blind trial. The trial was conducted in 13 specialized burn care centers in Germany. Adult Patients with 2b° or 3° burn injuries were included. Patients received state of the art burn care including obligatory split skin graft transplantation. Study medication was EPO or placebo every other day for 21 days. Between 12/08 and 06/14, 116 patients were randomized, 84 received study medication (EPO 45, Placebo 39). Primary endpoint analysis revealed inconclusive results, as only a minority of patients reached the primary endpoint [100% re-epithelialization: EPO: 23% (9/40); Placebo 30% (11/37)]. Several secondary endpoints such as SOFA score (morbidity), EPO level in blood and wound healing onset revealed clinical, and statistically significant results in favor of the EPO group. Adverse Events (AEs) and Severe Adverse Events (SAEs) were in expected ranges; AEs EPO: 80%, (36/45), Placebo: 77%, (30/39); SAEs EPO: 24%, (11/45), Placebo: 24%, (8/39). Out of 84 patients two died, one per group, thus mortality was lower than expected. Results (SOFA score) indicate a lower morbidity of the EPO group, suggesting pro-regenerative effects of EPO in burned patients. Higher EPO levels might influence the faster onset of re-epithelialization in the first 10 days of the treatment. Both effects could reveal new therapeutic options.

**Clinical Trial Registration:** ISRCT Number: ISRCTN95777824 and EudraCT Number: 2006-002886-38, Protocol Number: 0506.

## Introduction

Annually approximately one million adult patients receive specialized treatment for burn and scalding injuries, with an average of 180.000 fatal outcomes worldwide (World Burn Foundation, 2016; World Health Organization, 2016). Due to improved critical care treatment, and innovations in surgical technics, the survival rate of major burn injuries has improved during the last decades (Jackson, 1953; Singer and Dagum, 2008).

Cutbacks due to wound infections and excessive, mutilating scars are still numerous (Cohen, 2000). In burned patients like in all other critically ill and trauma patients, inflammatory states, wound infections, sepsis, and SIRS are the main course for fatal outcomes (Ottomann and Hartmann, 2004).

Challenges in improving burned patient survival and outcomes include activation of the body’s own regenerative capacities. This aims on reduction of wound infection risks, decreasing scar formation, and the systemic inflammatory answer to the burn trauma.

So far erythropoietin (EPO) and its effects were investigated in acute and chronic tissue damage in preclinical and a few clinical trials (Jelkmann, 2007; Brines and Cerami, 2008; Solling, 2012; Günter et al., 2013a). EPO and EPO-receptor synthesis and its anti-apoptotic, pro-angiogenic, pro- regenerative, anti-inflammatory, immunomodulatory and hypoxia/ischemia protective effects have been described in many tissues and organs (Brines and Cerami, 2008; Minnerup et al., 2009; Solling, 2012; Günter et al., 2013b). Previous publications using rodent models have described faster wound healing, higher quality of scars, prevention of secondary burn progression, higher levels of stem cell markers and higher amount of newly formed blood vessels in burn and scalding injuries or other wound models (Galeano et al., 2006; Tobalem et al., 2012; Giri et al., 2015). The erythropoietic response to EPO is markedly decreased in thermally injured patients (Still et al., 1995). In critically ill patients, EPO therapy is described to be safe and beneficial when combined with an adequate anti-thrombotic prophylaxis. Critically ill trauma patients revealed decreased mortality rates, hypothesized due to EPOs protective effects from hypoxemia/ischemia and mediation of local stress response (Corwin et al., 2007).

We used the previously mentioned findings of the pleiotropic effects of EPO as the basis for our hypothesis that EPO might reveal beneficial effects on wound healing, scar formation, morbidity and survival in patients with burn injuries.

Study objectives were to demonstrate a cytoprotective and regenerative effect of EPO in thermally injured patients in terms of reduced morbidity and mortality and to better understand the cellular mechanisms of EPO in Skin Graft Donor Sites (SGDS) and Second Degree Wounds (SDW).

## Materials and Methods

This clinical trial was prospective, placebo-controlled, randomized, double-blind, and conducted in thirteen specialized burn care centers throughout Germany. The trial was conducted according to globally accepted standards of good clinical practice in agreement with the Declaration of Helsinki and in keeping with local regulations. It had full approval of the designated ethics committees of all centers (leading ethic committee: University of Lübeck, Lübeck, Germany). The conduct of the trial was supported by three independent clinical research organizations: Münchner Studienzentrum, KKS Düsseldorf, and IFOM Köln.

Informed consent in written form had to be given before the first study-specific action either by patient’s themselves, patient’s legal representatives or via the “Heidelberger Verfahren” (Brückner et al., 2010). If the patient was included via the “Heidelberger Verfahren” or via the patient’s legal representative, patients were approached for written informed consent as soon as it was medically appropriate.

Patients received state-of-the-art burn and critical care treatment. Depending on the clinical situation of the individual patients this included analgosedation, respiration, escharectomy, antibiotic treatment, dialysis and other treatments. A split skin graft transplantation was obligatory, because the study wound was a defined SGDS. Therefore adult Patients with 2b° or 3° burn injuries were included, who needed split skin grafting. Study wounds had to be treated with a special polyurethane-foil dressing as described in detail in the study protocol and SOPs (Dornseifer et al., 2009).

Study medication was either 150 International Units (IU) EPO (NeoRecormon^®^ Multidose 50,000 IU, Roche, Grenzach-Wyhlen, Germany; off-label use) per kg body weight or a matched placebo (Günter et al., 2013a). Study medication was administered every other day subcutaneously for a maximum 21 days.

A central managing pharmacy acting independently from the principle investigator and the study centers was responsible for study drug blinding and study drug logistics. Blinding of the study personnel and the patients was ensured by institutional, logistical and geographical division of study pharmacy, centers and CRO.

Allocation to treatment group was done chronologically using a pre-defined randomization list, which was delivered to the pharmacy for blinding the study medication. This list was created using www.randomization.com using stratification per study center and a fixed block-size of 10 with allocation ratio 1:1.

The primary endpoint was the time until complete re-epithelialization of the defined SGDS. Biopsies were taken, and clinical assessments were performed at study days D 02, D 10, D 12, and D 16. Histological wound evaluation scoring included re-epithelialization, neovascularization, amount of granulation tissue, and number of inflammatory cells. The scoring system was modified from Sevimli-Gur et al. (2011) (Figures 2A–D).

Secondary endpoints were: The time until complete re-epithelialization of a defined type 2a° wound (SDW), and a defined split skin graft (TDW), daily controls of organ failure parameters (Sequential Organ Failure Assessment score = SOFA score), vital signs, laboratory controls (potassium, creatinine, glutamate-oxaloacetate-transaminase, gamma-glutamyl-transferase, white blood cell count, hemoglobin, cholinesterase, alkaline phosphatase, bilirubin, uric acid) and safety relevant parameters (red blood cell count, hematocrit, thrombocytes, international normalized ratio, partial thromboplastin time), packed red cell units transfused (PRCU), quality of scar formation (Vancouver scar scale) and assessment of quality of life (SF-36).

EPO levels in the peripheral blood were assessed at D 01 and at Ds 07, 14, 21, 28 and 42 of the study. EPO was assayed in duplicate in plasma samples by double-antibody sandwich enzyme-linked immunoassay (Quantikine^®^ IVD^®^ ELISA, R&D Systems, Minneapolis, MN, United States) following the manufacturer’s instructions. The inter-assay coefficient of variance was 11.2% (*n* = 14) at 8 mU/ml. According to the manufacturer’s information, the mean recovery is 100% and the sensitivity of less than 0.6 mU/ml. We analyzed EPO blood levels in 21 patients.

Concerning the Sequential Organ Failure Assessment score (SOFA score): As burn wounds are very painful, it is common to treat patients, with analgosedation for pain control. Therefore, we did not collect the neurological data for the SOFA Score (Glasgow coma scale), due to the fact that the analgosedation was not caused by neurological problems, instead, it was rather the result of the therapeutic intervention to treat the condition (Jones et al., 2009).

As quality control measures we had implemented a GCP conform monitoring and auditing program during planning, conduct and analysis of the trial. All of them were described in detail either in the trial protocol, trial SOPs or in the monitoring and statistical analysis plans. Safety endpoints included AEs, serious AEs (SAEs), suspected unexpected serious adverse reactions (SUSARs), laboratory test results and vital signs. The Data Safety Monitoring Board (DSMB) supervised the occurrence of AE, SAE, SUSAR and overall safety parameters. All SAEs were carefully evaluated by the coordinating study center documented in SAE forms and reported to the respective regulatory authority.

For statistical analysis we used GCP conform and validated software: nQuery Advisor 7.0, SASS Version 9.4, and R)^1^.

Planned Sample Size: Under conventional treatment, complete re-epithelialization was assumed to occur after a mean of 10–15 days with a standard deviation (SD) of about 6 days, depending on donor site and patients’ general condition. We expected about 70% of re-epithelialization to lay within ±6 days from the average expectation assuming a normal distribution of data. It was also expected that EPO treatment can reduce total time to heal by at least 4 days. In addition 4 days are in our opinion the smallest number which would make a considerable clinical difference for the patient and treatment success. A sample size of 49 in each group would, therefore, have 90% power to detect a difference in means of 4 days, assuming that the common SD is 6 days using an independent samples *t*-test with a 0.05 two-sided significance level (nQuery Advisor 7.0) Rabbels (2004), and Dornseifer et al. (2009).

The intention-to-treat (ITT) analysis set contained all randomized patients with results attributed to the treatment group that they were randomized to and who received at least one dose of study medication. The per-protocol (PP) set contained all patients of the ITT set except for those with major protocol violations until and including Day 21.

The distribution of continuous data is presented by mean ± SD. In case of severe deviations from the normal distribution, median and interquartile range (IQR) are given instead. Categorical data are presented by absolute and relative frequencies.

The two treatment arms were compared and tested for the superiority of EPO over placebo with respect to time until complete re-epithelialization of SGDS. Due to non-normality of the data, it was decided in the blinded data review meeting (BDRM) to apply a two-sided van Elteren test, instead of a *t*-test, with ABSI score (<7 vs. ≥7) as stratum variable for analysis on a confirmatory 5% significance level in the ITT population. Since wound healing is recorded as ordinal data in the CRF (values: 0; 1–25, 26–50, 51–75, 76–99, and 100%), the last recorded value for re-epithelialization was compared between treatment groups using absolute and relative frequencies of each re-epithelialization value and an exploratory Mann-Whitney *U* test as sensitivity analysis on an two-sided 5% significance level.

All between-group comparisons of secondary endpoints were performed on the ITT and PP groups by Mann-Whitney *U* tests. Likewise, sensitivity analyses of the primary endpoint were performed by repeated application of the van Elteren test in subgroups. All tests of secondary endpoints were performed on exploratory two-sided 5% significance levels.

The “worst-case scenario” approach was used for the analysis of the primary endpoint. Missing values in the EPO arm were set to 31 days. In the placebo arm, it was set individually on the last day the endpoint and was investigated for a patient with a limiting maximum of 16 days. This concerns only missing values of the primary endpoint until and including day 16. Other missing values were not imputed. One unblinded patient was managed the same way.

For more detailed information please compare our protocol publication (Günter et al., 2013a).

## Results and Discussion

We screened 3292 patients, 116 patients who met eligibility criteria were randomized into the trial (see Table 1). 32, who did not receive study medication due to discontinuation of study participation upon personal request or by request of their legal representative, before the first study drug application ensued. 84 participants, who received the study medication at minimum once and were included into the ITT population (EPO 45, Placebo 39). 53 patients were treated as described in the protocol (EPO 29, Placebo 24) and were included into the per protocol (PP) population. Thirty-six patients missed the follow ups, and one patient was unblinded.

**Table 1:** In- and exclusion criteria of the trial.

**Inclusion Criteria:**
• 3° or 2b° burn and scalding injuries obligate, 2° burn and scalding injuries optional, only required if no 3° injury available and operations including split skin harvesting and grafting are required for 2b° injury • Men and women, age ≥18 and ≤ 75 years • Secure contraception (http://www.ema.europa.eu/docs/en_GB/document_library/Scientific_guideline/2009/09/WC500002941.pdf)
**Exclusion Criteria:**
• Admission later than 24 h after injury • Hematological disorders (anemia, lymphoma, leukemia, inborn coagulation diseases) • Pregnancy or breast-feeding • Estimated survival shorter than 1 week (abbreviated burn severity index (ABSI) >12) in patients older than 40 years of age • Body weight <50 kg or >110 kg • Total burn surface area involved less than 60% in patients older than 40 years of age. • In patients below 40 years of age no limitation of maximum burned body surface and no limitation of ABSI score will be considered • Upper lateral thighs of both legs thermally injured • Subject is the investigator or any sub-investigator, research assistant, pharmacist, study coordinator, other staff or relative thereof directly involved in the conduct of the protocol • Subject is unlikely to comply with protocol, e.g., uncooperative attitude, inability to return for follow-up visits, and unlikelihood of completing the study • Treatment with any investigational product in the last 12 months before study entry • Treatment with any immunosuppressive therapy, cancer-related chemotherapy or radiation therapy in the past 12 months • History of hypersensitivity to the investigational products • Likelihood of requiring treatment during the study period with drugs not permitted by the clinical study protocol • Clinically relevant cardiovascular (cardiac infarction, coronary heart disease (CHD), thromboembolic disease, thromboembolic events shortly before admission), hepatic (Child B or C liver disease), endocrine [morbid obesity (BMI >40)] or systemic (cancer) disease • Epileptiform diseases • Phenylketonuria • HIV disease, AIDS • Informed consent missing

**FIGURE 1:**
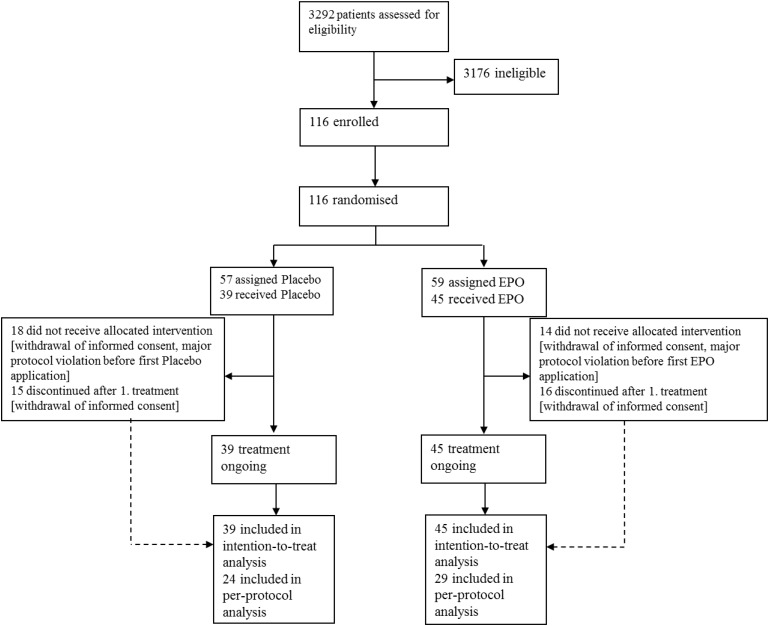
Patient flow. Legend: Enrolment: patients with burn and scald injuries who met all inclusion criteria and none of the exclusion criteria were included. Allocation: centralized randomization (stratified by study center) patients were randomly allocated to the verum- or the placebo-treated group using a variable block size, patients received max. 11 study drug administrations. Follow-up: at Day 42, after 6 months, and 12 months past inclusion. Analysis: ITT analysis set: received at least one dose of study medication. PP set: received at least one dose of study medication but displayed major protocol violations until and including Day 21.

Five patients prematurely stopped participation in the study upon personal request, but after minimum one study drug application. For 13 patients other reasons for discontinuation of participation were additionally documented in the CRF. Two patients (one in each group) died. (Patients flow, see Figure 1)

Placebo and EPO treated patients in the ITT population had a mean (± standard deviation) age of 46.6 ± 15.0 years and 48.5 ± 16.2 years, respectively. A clear predominance of males suffering from burn and scalding injuries in Germany is confirmed by 73% (EPO: 33/45) and 85% (Placebo: 33/39) male patients. ABSI Score distribution in the ITT group: we found that 40% (34/84) of the patients had an ABSI Score <7 (EPO: 47% (21/45), Placebo: 33% (13/39)).

Subpopulations were well balanced between EPO and placebo groups regarding typical baselines variables of patients with burn and scalding injuries (see Tables 2, 3, 8, 9, and Supplementary Material).

**Table 2:** Baseline characteristics of ITT population.

Baseline characteristics (ITT)	EPO (*n* = 45)	Placebo (*n* = 39)	Sum (*n* = 84)
Age – years (SD)	48.5 ± 16.2	46.6 ± 15.0	
Age ≥60 years – no. (%)	8 (18%)	10 (26%)	18 (21%)
Female	12 (27%)	6 (15%)	18 (21%)
ABSI score ≥7 – no. (%)	24 (53%)	26 (67%)	34 (40%)
TBS-Sum – % (SD)	24.9 ± 11.7	26.6 ± 13.3	
AEs – no. (%)	36 (80%)	30 (77%)	66 (79%)
SAEs – no. (%)	11 (24%)	8 (21%)	19 (23%)
Death – no. (%)	1 (2%)	1 (2%)	2 (2%)

*For the ITT population data are mean and standard deviation or n (%). ABSI score = Abbreviated burn severity index score = The ABSI Score describes the severity of the burn or scalding injury and the prognosis of the patient. The lower the ABSI score the better is the prognosis of the patient. An ABSI over 7 relates to a serious threat of life, with a probability of survival between 70 and 0%. TBS-Sum = total body surface thermally injured in %. AEs = adverse events: number of patients with AEs, SAEs = serious adverse events: number of patients with SAEs. Abbreviated Burn Severity Index (Tobiasen et al., 1982).*

**Table 3:** Concomitant diseases and injuries of ITT population (ICD-10 codes).

Classification	Diseases	Counts	EPO	Placebo
**Certain infectious and parasitic diseases**		2	1	1
	Hepatitis B	1	0	1
	Herpes zoster neuralgia	1	1	0
**Neoplasms**		2	0	1
	Bronchial carcinoma	1	0	1
	Carcinoma of floor of mouth	1	0	1
**Diseases of blood and blood-forming organs**		1	1	0
	Chronic iron deficiency	1	1	0
**Endocrine, nutritional and metabolic diseases**		16	11	5
	Diabetes mellitus type 2	6	5	1
	Hypercholesterolemia	3	3	0
	Hyperthyreosis	1	0	1
	Hyperuricaemia	2	1	1
	Hypothyreosis	2	1	1
	Hypouricaemia	1	1	0
	Idiopathic unconjugated hyperbilirubinaemia (Gilbert’s syndrome)	1	0	1
**Mental and behavioral disorders**		18	11	7
	Alcohol abuse	4	2	2
	Delirium	1	0	1
	Depressive disorders	3	3	0
	Nicotine abuse	4	3	1
	Obsessive-compulsive disorder	1	0	1
	Oligophrenia	1	0	1
	Psychosis	3	3	0
	Schizophrenia	1	0	1
**Diseases of the nervous system**		4	2	2
	Epilepsy (symptomatic)	2	1	1
	Intercostal neuralgia	1	1	0
	Parkinson disease	1	0	1
**Diseases of the eye and adnexa**		2	2	0
	Cataract	1	1	0
	Conjunctivitis	1	1	0
**Diseases of the ear and mastoid process**		1	0	1
	Hearing loss (hypacusis)	1	0	1
**Diseases of the circulatory system**		27	15	12
	Angina pectoris (unstable)	1	1	0
	Arterial hypertension	18	11	7
	Cardiac arrhythmia	3	2	1
	Cerebral infarction (stroke)	1	0	1
	Coronary heart disease	2	0	2
	Thrombosis (lower-leg)	1	0	1
	Unspecified right bundle-branch block (Brugada-Syndrome)	1	1	0
**Diseases of the respiratory system**		8	6	2
	Atopic asthma	1	1	0
	Bronchial asthma	2	1	1
	COPD	3	2	1
	Non-atopic asthma	1	1	0
	Reinke’s edema	1	1	0
**Diseases of the digestive system**		1	0	1
	Duodenal ulcer	1	0	1
**Diseases of the skin and subcutaneous tissue**		1	1	0
	Psoriasis vulgaris	1	1	0
**Diseases of the musculoskeletal system and connective tissue**		3	2	1
	Displacement intervertebral disc (prolapse)	1	1	0
	Polymyalgia rheumatica	1	1	0
	Rheumatism	1	0	1
**Diseases of the genitourinary system**		1	1	0
	Prostatic hyperplasia	1	1	0
**Injury, poisoning and certain other consequences of external causes**		14	10	4
	Blunt abdominal trauma	1	1	0
	Cerebral contusion with bleeding	1	1	0
	Cerebral edema	1	1	0
	Fracture of calcaneus	2	1	1
	Fracture of humerus	1	0	1
	Fracture of lower leg, including ankle	3	2	1
	Fracture of metatarsal bone 5	1	0	1
	Fracture of pelvis (s/p)	1	1	0
	Pulmonary contusion	1	1	0
	Rupture of rotator cuff	1	1	0
	Traumatic compartment syndrome of right lower extremity	1	1	0
**Factors influencing health status and contact with health services**		6	4	2
	History of (lower-) leg amputation (s/p)	1	1	0
	History of lung lobectomy	1	0	1
	Nephrectomy	1	0	1
	Penicillin allergy	1	1	0
	Presence of xenogeneic heart valve (aortic)	2	2	0

*COPD = Chronic Obstructive Pulmonary Disease.*

The primary outcome measure, re-epithelialization of the study site until day 16, revealed inconclusive results, as only a minority of patients reached the primary endpoint. Apart from the two patients who died, the primary endpoint was unavailable for 64 of 84 patients in the ITT set and had to be imputed for the analysis of the primary endpoint. Consequently, the result is dominated by the imputation of missing values according to the “worst-case scenario”. In fact, using the Van Elteren test, there was a statistically significant difference in the ITT population with a median of 31 days in the EPO group and 16 days in the placebo group (*p* < 0.001). In the PP population (EPO: 29, Placebo: 24), this difference was 31 days to 16 days (*p* < 0.001), respectively.

Sensitivity analysis was performed to investigate the number of cases and the percentage of re-epithelialization reached. Overall looking at the concrete numbers and the percentage of re-epithelialization we cannot find any relevant differences (see Table 4).

**Table 4:** Descriptive statistics (absolute and relative frequencies) re-epithelialization of split skin graft donor site of ITT population.

	26–50%	51–75%	76–99%	100%	Sum
**Split skin graft Donor site re-epithelialization of D 16**					
EPO - no. (%)	1 (2%)	8 (20%)	22 (55%)	9 (23%)	40
Placebo - no. (%)	2 (5%)	4 (11%)	20 (54%)	11 (30%)	37
Sum - no. (%)	3 (4%)	12 (16%)	42 (54%)	20 (26%)	77

*ITT population data are presented as absolute numbers and in %.*

**FIGURE 2:**
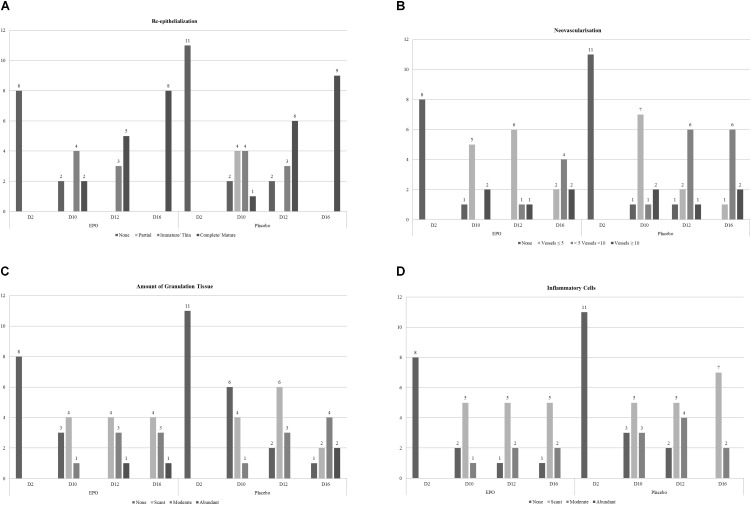
Wound healing evaluation scale. Legend: We analyzed histology’s in 21 patients (EPO: 10, Placebo: 11), but 2 EPO patients had too many missing biopsies, so we excluded them from analysis. The bars show absolute patient numbers. The histologic features were assessed by the same specialist (blinded to the treatment) in paraffin-embedded sections using hematoxylin/eosin stains under light microscopy at a magnification of 5X to 10X. Histological wound evaluation scoring included: **(A)** re-epithelialization (0 = none; 1 = partial; 2 = complete, but immature/thin; 3 = complete and mature), **(B)** neovascularization (0 = none; 1 = up to 5 vessels/high-powered magnification field [HMF]; 2 = 6–10 vessels/HMF; 3 = > 10 vessels/HMF), **(C)** amount of granulation tissue (0 = none; 1 = scant; 2 = moderate; 3 = abundant), and **(D)** inflammatory cells (0 = none; 1 = scant; 2 = moderate; 3 = abundant). The scoring system was modified from Sevimli-Gür et al. (2011).

H&E histological assessments and the evaluation of the wound healing evaluation score revealed large inter-individual differences, between the two groups results were rather inconclusive (see Figure 2).

**FIGURE 3:**
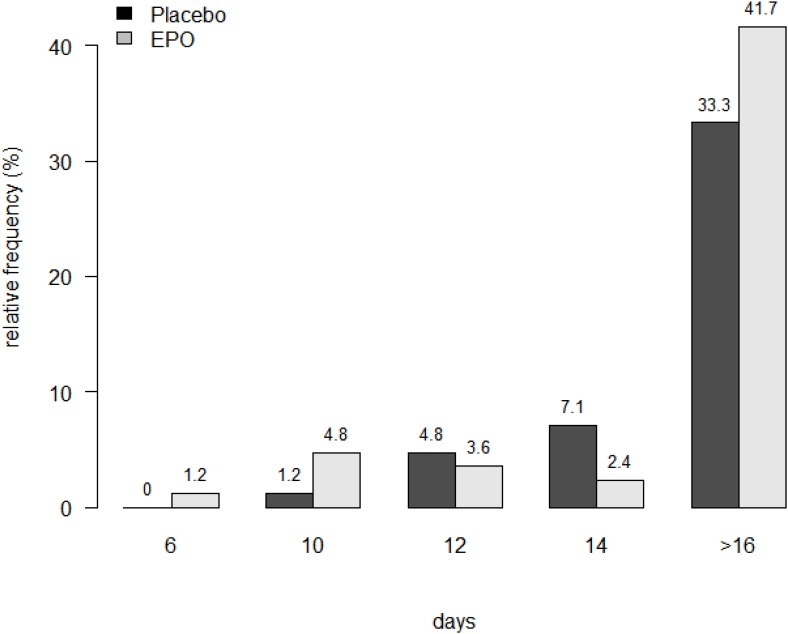
Detailed Analysis of Re-epithelialization of Study Site at Days: 6, 10, 12, 14 and 16. Legend: ITT population data are presented in % of patients with a complete healed study site at the respective days. Remark the high number of not healed study sites at D 16.

A detailed sub-analysis of patients with completely re-epithelialized study sites at days: 6, 10, 12, 14, and 16 displayed more completely re-epithelialized study sites in the EPO group at days 6 and 10, with a decrease at day 12 (Figure 3). In addition, the results revealed a majority of incomplete re- epithelialized study sites in both treatment groups (ITT: EPO: 77% (31/40), Placebo: 70%, (26/37); PP: EPO: 82% (23/28), Placebo: 67% (16/24)) (see Figure 3).

**FIGURE 4:**
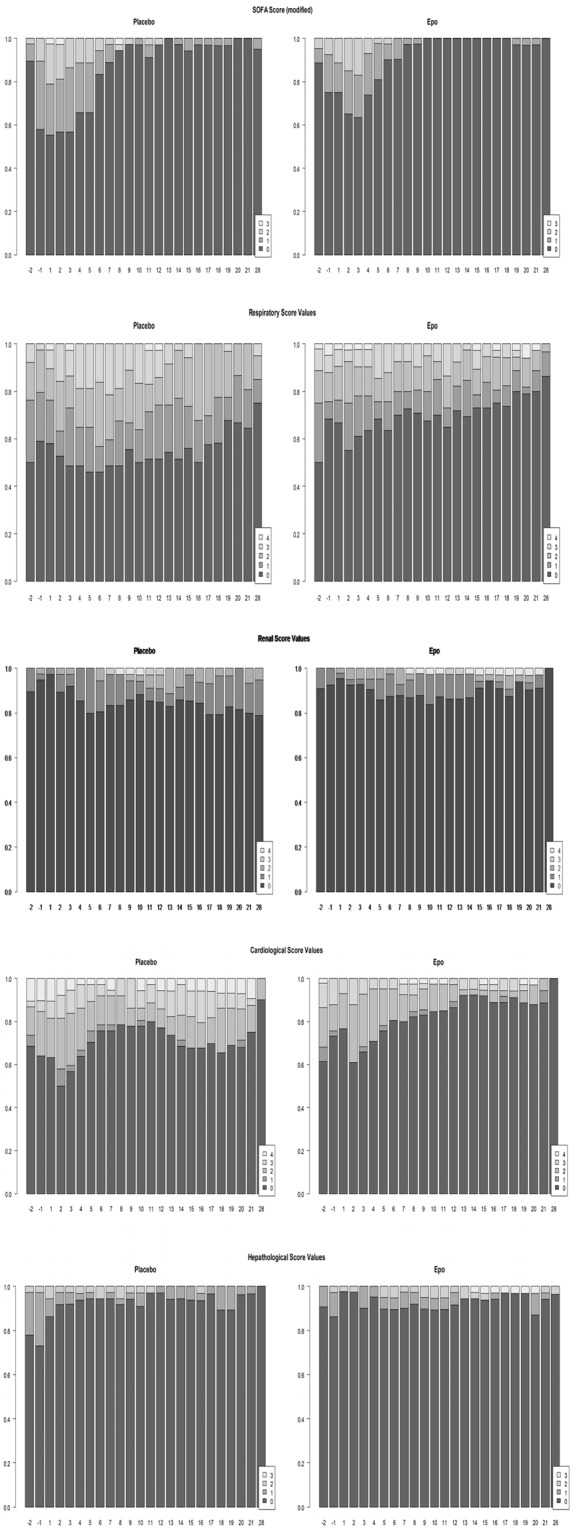
Modified SOFA score results. 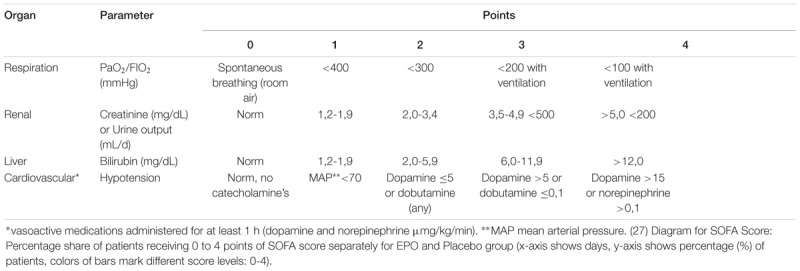

The analysis of the SOFA score, especially the sub analysis of the source data of the cardiac and the respiratory SOFA score revealed results in favor of the EPO group in the ITT (EPO: *n* = 41, Placebo: *n* = 39) and PP (EPO: *n* = 25, Placebo: *n* = 24) populations. At admission both groups displayed comparable SOFA scores, but after study drug administration the EPO group displayed lower SOFA score results with lasting effect (see Figure 4).

**FIGURE 5:**
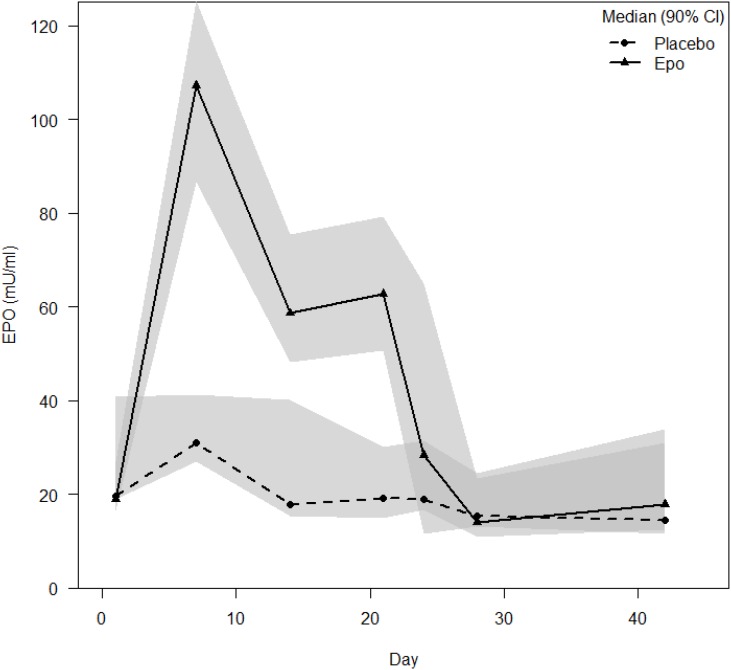
EPO levels in the peripheral blood. Legend: Plasma EPO level data are displayed in SI units’ median with two-sided 90% confidence intervals.

During the treatment, EPO group patients displayed higher EPO levels than placebo group patients (EPO: 8, Placebo: 11). Noticeable is the high peak in the EPO group (Median 112.63 mU/ml) and much lower but detectable also in the placebo group (Median 38.1 mU/ml) at D 7 and the decrease to the plateau phase at D 14 (EPO: Median 65.0 mU/ml; Placebo: Median 30.9 mU/ml) and D 21 (EPO: Median 67.2 mU/ml, Placebo: Median 23.9 mU/ml) without changes in the dosing regimen (see Figure 5).

**FIGURE 6:**
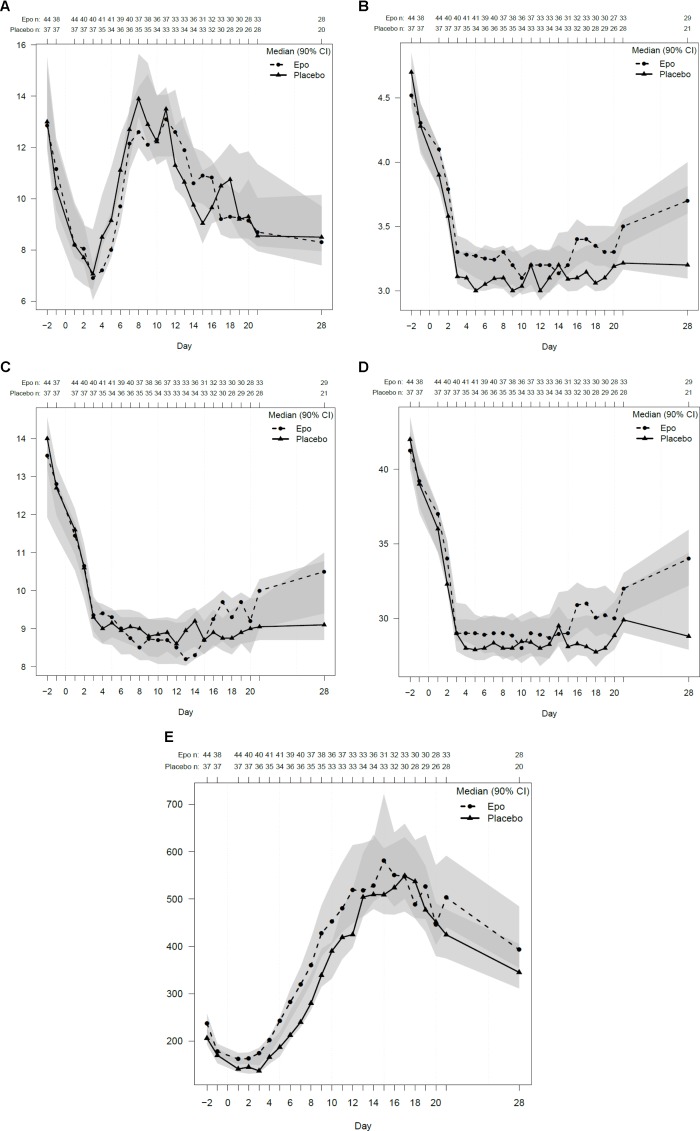
Laboratory control results. Legend: Laboratory control data are displayed in SI units’ median with two-sided 90% confidence intervals. **(A)** White Blood Cell Count (/nl). **(B)** Red Blood Cell Count (/pl). **(C)** Hemoglobin (g/l). **(D)** Hematocrit (%). **(E)** Thrombocytes (/nl).

There were differences regarding the results of the laboratory analysis between the two groups as well as inter-individual differences, but these findings did not constitute a statistically significant difference, although there might be a clinically relevant difference (see Figures 6A–E). For example regarding the creatinine values (data not displayed) compared with the SOFA score results, there seemed to be clinically relevant findings. Red blood cell counting’s as well as hemoglobin and hematocrit and thrombocyte values seemed to display a trend to higher values in the EPO group (Marck et al., 2013).

We detected differences regarding the total amount of transfused packed red cell units (PRCU) (EPO: *n* = 18, Median = 2, Mean = 6.9; Placebo: *n* = 12, Median = 4, Mean = 7.1), which corresponded to the clinical situation of the individual patient (EPO: range = 0–65; Placebo: range = 0–35) (see Table 5).

**Table 5:** Packed red cell units transfused.

	EPO (ITT=43/ PP=27)	Placebo (ITT=38/PP=23)
**Summary of Data on Red-Cell-Transfusion (ITT and PP)**		
Patient receiving min. one transfusion – no. (%)	18 (42%) / 9 (33%)	12 (32%) / 4 (17%)
Units transfused per patient – no.	39.1/23.5	43.2/27.9
Mean +/−SD	6.9 ± 11.9/6.7 ± 7.8	7.1 ± 9.0/9.3 ± 10.1

*ITT and PP population data are presented as absolute numbers in % and mean and SD.*

In accordance to inconclusive results of the primary endpoint, the analysis of wound healing of the SDW and the TDW revealed inconclusive results as well (see Tables 6, 7).

**Table 6:** Analysis of secondary outcome: SDW Re-epithelialization.

	26–50%	51–75%	76–99%	100%	Sum
**SDW re-epithelialization (ITT, n=62 documented cases)**					
EPO - no. (%)	4 (12%)	5 (16%)	14 (44%)	9 (28%)	32
Placebo - no. (%)	2 (6%)	3 (10%)	14 (47%)	11 (37%)	30
Sum - no. (%)	6 (10%)	8 (13%)	28 (45%)	20 (32%)	62

*ITT population data are presented as absolute numbers and in %.*

**Table 7:** Analysis of secondary outcome: TDW Re-epithelialization.

	26–50%	51–75%	76–99%	100%	Sum
**Skin graft re-epithelialization (TDW) (ITT, n=76 documented cases)**					
EPO - no. (%)	4 (10%)	7 (17%)	17 (43%)	12 (30%)	40
Placebo - no. (%)	2 (6%)	4 (11%)	13 (36%)	17 (47%)	36
Sum - no. (%)	6 (8%)	11 (14%)	30 (40%)	29 (38%)	76

*ITT population data are presented as absolute numbers and in %.*

Scar formation revealed after 6 (total 45; EPO 22, Placebo 23 patients) and 12 (total 36; EPO 18, Placebo 18 patients) month inter-individual differences but none between groups. Likewise, the differences were indifferent with respect to the quality of life after 6–12 months (data not displayed).

Two patients died during the trial; due to multi-organ failure (sepsis/Placebo) and cardiac arrest (electro-motoric decoupling/EPO). Relationship to EPO/Placebo was deemed not possible.

A total of 214 AEs were reported in 66 of the patients [EPO: 80% (36/45), Placebo: 77% (30/39)], 24 AEs were deemed severe. Most AEs concerned laboratory controls (investigations) (63 AEs, CRP increase and changes in liver parameters), blood and lymphatic system disorders (49 AEs, leukocytosis, leukopenia, and thrombocytosis) or infections (32 AEs, wound infections, pneumonia or urinary tract infections) (see Table 8).

A total of 24 SAEs were reported in 19 patients (EPO: 24% (11/45), Placebo: 21% (8/39)). Five of the 24 documented SAEs fife were classified as possibly related (SARs). For 19 SAEs a relationship was ruled out. Twenty-two/24 SAEs resolved without lasting damage to the patients’ health (see Table 8).

**Table 8:** Adverse events and severe adverse events.

	EPO	Placebo	Sum
**AEs (terms reported)**			
Blood and lymphatic system disorders	27	22	49
Cardiac disorders	0	1	1
Gastrointestinal disorders	1	0	1
General disorders and administrative site conditions	9	8	17
Hepatobiliary disorders	1	4	5
Infections and infestations	16	16	32
Injury, poisoning and procedural complications	0	1	1
Investigations (laboratory values)	41	22	63
Metabolism and nutrition disorders	2	3	5
Musculoskeletal and connective tissue disorders	0	1	1
Nervous system disorders	1	0	1
Psychiatric disorders	1	1	2
Renal urinary disorders	0	3	3
Respiratory, thoracic and mediastinal disorders	4	4	8
Skin and subcutaneous tissue disorders	4	1	5
Surgical and medical procedures	1	0	1
Vascular disorders	1	1	2
Sum^∗^	109	88	197
**SAEs (Terms reported)**			
Blood and lymphatic system disorders	3	0	3
Cardiac disorders	0	1	1
Infections and infestations	6	4	10
Injury, poisoning and procedural complications	1	0	1
Investigations (laboratory values)	0	1	1
Nervous system disorders	2	0	2
Renal urinary disorders	1	3	4
Respiratory, thoracic and mediastinal disorders	4	3	7
Vascular disorders	0	1	1
Sum^∗∗^ of Terms	17	13	30

*For the listings and coding of AEs and SAEs we used MedDRA Version 15.1. ^∗^Number of reported AEs = 214, Terms of diseases within AEs =224, without double counting of same AE-Term within one patient = 197. ^∗∗^ Reported SAEs =24, Terms within SEAs=30.*

**Table 9:** Concomitant medication of ITT population.

Classification of drugs	Number of patients	Pharmaceutical Name
Antidepressant	17	Amitriptylin, Citalopram, Escitalopram, Mirtazapin, Venlafaxin, Trimipramin
Anticonvulsants	10	Phenytoin, Pregabalin (Lyrika)(9), Primidon
Benzodiazepine	62	Brotimazol, Clorazepam, Diazepam, Flunitrazepam, Lorazepam, Lormetazepam, Midazolam, Nitrazepam, Oxazepam, Zolpidem, Zopiclone
Muscle Relaxant	10	Pancuronium, Rocuronium
Narcotics	52	Etomidat, Isofluran, Ketamin, Propofol
Neuroleptics	18	Haloperidol (Sedierung), Olanzapin, Promethazin, Quetiapin, Risperidon
NSAIDs	70	Dexketoprofen, Diclofenac, Etoricoxibum, Ibuprofen, Metamizol, Paracetamol
Opioid (WHO clas. 1+2)	56	Dihydrocodin, Pethidin, Piritramid, Tilidin, Tramadol,
Opioid (WHO clas. 3+4)	73	Buprenorphin, Fentanyl, Morphin, Oxycodon, Remifentanil, Sufentanil, Tapentadol
Diuretics	46	Furosemid, HCT, Spironolakton, Triamteren, Torasemid
Vasopressors	43	Adrenalin, Dobutamin, Dopamin, Noradrenalin
Sympathomimetics	18	Salbutamol
Further cardio-vascular Medicaments	55	Acetysalicysäure, Amiodaron, Amlidipin, Clonidin (Schmerz), Atropin, Bisoprolol, Digitoxin, Enalapril, Metoprolol, Nifedipin, Olmesartan, Ramipril, Simvastatin, Sotalol,Telmisartan, Theophyllin, Urapidil, Verapamil,
Insulin	36	
Glucocorticoids^∗∗^	14	Beclometason dipropionat, Budesonid, Hydrocortison, Prednisolon
**Antibiotics, Antimycotics, Virostatics**		
Carbapeneme	23	Doripenem(1x), Imipenem, Meropenem
Cephalosporin	34	Cefazolin, Cefuroxim, Ceftazidim, Cefotaxim, Ceftriaxon, Cefepim,
Chino lone	22	Ciprofloxacin, Levofloxacin, Moxifloxacin
Glycopeptide	10	Vancomycin
Penicillin	45	Ampicillin, Flucloxacillin, Penicillin, Piperacillin/Tazobac/Combactam
Further Antibiotics	20	Amikacin+Gentamicin, Clindamycin, Daptomycin, Erythromycin, Fosfomycin, Linezolid, Metronidazol, Mupirocin, Polymyxin^∗^Colomycin, Tigecyclin
Antimycotics	11	Caspofungin, Clotrimazol, Fluconazol, Nystatin, Voriconazol
Virostatics	3	Aciclovir

*^∗^Indication: Diabetes mellitus II, Hyperglycemia. ^∗∗^Way of Application: local, systemic; Indication: Sepsis, Infection.*

There were five SARs reported in the trial (3 thrombocytosis, 1 deep vein thrombosis, 1 ischemic cerebral infarction). There was no SUSAR reported in the trial.

## Discussion

To our knowledge, this is the first randomized, placebo-controlled trial investigating EPO for the treatment of wound healing in patients with burn and scalding injuries.

The results regarding morbidity (SOFA-Score), wound healing over time, and mortality justifies further research.

The statistically significant result regarding the primary endpoint is probably a direct result of the infelicitous study design and the statistical analysis using the “worst case scenario”.

Regarding the more detailed analysis: wound healing over time, we observed faster re-epithelialization in the EPO group at days 6 to 10 ending at day 12. Publications of rodent models describe a faster onset of re-epithelialization in the EPO group with impairment of wound healing after repetitive EPO applications. The higher viscosity of the blood due to increased hematocrit values in the rodent models could explain this phenomenon. However, our patients did not display increased hematocrit values. Comparing wound healing over time and the EPO levels, we observed a fast onset of wound healing, which corresponded to the high EPO levels during the first 7 days. This might indicate an influence of higher EPO levels onto wound healing. The dependency of no-haematopoietic effects of EPO onto dosing, especially onto high EPO concentration has been published before (Sorg et al., 2009).

In publications about the pharmacokinetics of EPO in healthy volunteers and animal models comparable progressions of EPO levels were described. McMahon et al. (1990) reported plasma levels of EPO in healthy volunteers examined after repetitive injections, whereby the subjects showed a lower serum EPO concentration at day 10 compared to day 1. Levine et al. (Levine et al., 1989) suggested an increased rate of EPO metabolism resulting from high EPO levels, comparable to the effects seen in chronic anemia (Sherwood et al., 1986).

In pre-clinical studies, an inhibition of EPO production and inadequately low endogenous EPO levels due to septic conditions occurred (Jelkmann, 1998). So far, we could not find any evidence which directly described the decrease of exogenous EPO levels during treatment by increasing levels of inflammatory cytokines. But our findings are consistent with publications describing decreasing levels of EPO in chronic renal failure (Brockmöller et al., 1992). In an animal study, primates received EPO treatment and an exchange blood transfusion to imitate an acute bleeding and a sham laparotomy. With increasing white blood cell counts, the EPO levels in the primate model decreased as well (Levine et al., 1989). This might be due not only to an EPO production inhibition but also to an EPO consuming process caused by the inflammatory state of the organism. Analyzing the results of the SOFA Score, and herein especially the basic data of the respiratory- and the cardiac-system, revealed that the EPO group displayed a lower score and therefore a lower morbidity than the control group at multiple time points. This is a new finding adding onto the previously described lower mortality in critically ill patients (Corwin et al., 2007). It supports the hypothesis of anti-apoptotic, cyto- and tissue protective effects of EPO taking place in critically ill/burned patients (Brines and Cerami, 2008; Rocha et al., 2015).

Regarding the present ABSI scores, we would have expected approximately 30% fatal outcomes (Doyle, 1989). Importantly, however, we detected a lower mortality rate in both groups. A specific re-evaluation of the ABSI Score and its prognostic value in different clinical settings seems to be advisable.

The results regarding the re-epithelialization of the study wounds might, besides other reasons, be explained with the timing of the trial. Patients received the first study medication application at the earliest of 60 h after trauma. At this point of time, it was too late for short-term effects of EPO, such as prevention of secondary burn progression or subdermal-plexus thrombosis, NO release optimizing tissue perfusion, as well as anti-apoptotic effects (Tobalem et al., 2012). Only long-term effects, increase in skin perfusion, angiogenesis, stem cell mobilization, and stimulation of certain growth factors could be expected (Creutzig et al., 1990; Bader and Machens, 2010; Günter et al., 2013b).

The amount of transfused PRCU is in line with previous publications of clinical trials (Still et al., 1995; Corwin et al., 2007). An explanation for this effect is to be found in a recent publication using a mice model which revealed a shift in the direction of producing predominantly lymphatic cell lines after burn trauma, while erythropoiesis is suppressed (Posluszny et al., 2011).

Frequency and severity of the observed laboratory results (Stohlawetz et al., 2000), AEs and SAEs were in an even better range than to be expected regarding the ABSI scores of our patients, this finding is in concordance with our results regarding the SOFA score.

In our patient population, a high proportion of patients discontinued study participation prematurely upon personal request. This might be explained by the subjective impairment of general health status after dermal thermal trauma in many patients and with the “Heidelberger Verfahren”.

## Conclusion

Regarding pleiotropic EPO-effects, a discrepancy exists between the very promising results of preclinical investigations (Brines and Cerami, 2008) and the results of clinical investigations (Lundy et al., 2010; Solling, 2012). This EPO-paradox might relate to different timing and dosing regimens used. The dosage of EPO used in pre-clinical studies was frequently supra-physiological and therefore the described EPO effects could not be repeated with lower dosages used in the clinical trials (Jelkmann and Elliott, 2013). In animal studies, the timing of the EPO application was often in a very close time frame to the trauma, sometimes even before the trauma (Jelkmann, 1998; Saray et al., 2003). For obvious reasons such application regimes are not transferable into the clinical setting of burn and scalding injury treatment.

Never the less, SOFA score results indicate a lower morbidity of the EPO group, suggesting a pro-regenerative effect of EPO in burned patients. The faster onset of re-epithelialization within the EPO group might be influenced by higher EPO levels. Both effects could reveal new therapeutic options for remaining unmet clinical needs.

## Data Availability Statement

The [Clinical Trial] data used to support the findings of this study are included within the article.

Previously reported [Protocol Publication] data were used to support this study and are available at [doi: 10.1186/1468-6708-14-124]. These prior studies (and datasets) are cited at relevant places within the text as reference Günter et al. (2013a).

## “Epo in Burns” Study Group

Dunda S, Grieb G, Wolter T, Pallua N, Namdar T, Ottomann C, von Wild T, Mailänder P, Thamm OC, Dornseifer U, Lonic D, Ninkovic M, Siemers F, Sievers R, Reichert B, Ernert C, Steen M, Schaller HE, Otte M, Hartmann B, Ryu S-M, Pierson T, Ohmann C, Neugebauer E.

## Author Contributions

AB and H-GM designed the trial and protocol with assistance from Edmund Neugebauer (IFOM Köln). CG, SE-S, SG, FI, WJ, H-GM, and AB conducted the trial and collected the data in cooperation with Münchner Studienzentrum. CG managed, analyzed, and interpreted the data in cooperation with FI, WJ, and AH, Institute of Medical Statistics and Epidemiology (IMSE), Münchner Studienzentrum, and Koordinierungszentrum für Klinische Studien Düsseldorf. CG wrote the manuscript and is responsible for the final version. AH wrote the statistics part. CG, AH, WJ, FI, SE-S, H-GM, SG, and AB contributed to data interpretation, revision of the manuscript, as well as review of accuracy, and completeness of the reported data. CG had full access to all data congregates in the study and made the final decision to submit the manuscript for publication.

## Conflict of Interest Statement

AB holds several patents concerning EPO in several pharmaceutical applications. WJ received grants from “Amgen” as a speaker for several scientific meetings.

The remaining authors declare that the research was conducted in the absence of any commercial or financial relationships that could be construed as a potential conflict of interest.

## Footnotes

1 https://www.r-project.org/
