# Supplementary material for: A Randomized Controlled Trial: Regenerative Effects, Efficacy and Safety of Erythropoietin in Burn and Scalding Injuries
Source: Front Pharmacol. 2018 Oct 31;9:951. doi: 10.3389/fphar.2018.00951 (PMC6220439; doi:10.3389/fphar.2018.00951)
Supplement: Supplementary file 1 [file Data_Sheet_1.docx]

**As additional online information:**

**Laboratory Control Results**

Legend: Laboratory control data are displayed in SI units’ median with two-sided 90% confidence intervals.

**Potassium (mmol/l)** **as additional online information**

**Creatinine (µmol/l)** **as additional online information**

**Glutamate-Oxalacetate-Transaminase (U/l)** **as additional online information**

**Cholinesterase (kU/l)** as additional online information

**Alkaline Phosphatase (U/l)** as additional online information

**Bilirubin (mg/dl) as additional online information**
